# Current approaches on non-invasive prenatal diagnosis: Prenatal genomics, transcriptomics, personalized fetal diagnosis

**DOI:** 10.4274/tjod.26817

**Published:** 2014-12-15

**Authors:** Tuba Günel, Mohammad Kazem Hosseini, Ece Gümüşoğlu, Görkem Zeybek, İsmail Dölekçap, İbrahim Kalelioğlu, Ali Benian, Hayri Ermiş, Kılıç Aydınlı

**Affiliations:** 1 İstanbul University, Faculty of Science, Department of Molecular Biology and Genetics, İstanbul, Turkey; 2 Çanakkale Provincial State Hospital, Clinic of General Obstetrics and Gynecology, Çanakkale, Turkey; 3 İstanbul University İstanbul Faculty of Medicine, Department of Gynecology, İstanbul, Turkey; 4 İstanbul University Cerrahpaşa Faculty of Medicine, Department of Gynecology, İstanbul, Turkey; 5 Medicus Health Center, İstanbul, Turkey

**Keywords:** Genomics, Non-invasive, Prenatal diagnosis, fetal personalized medicine

## Abstract

Recent developments in molecular genetics improved our knowledge on fetal genome and physiology. Novel scientific innovations in prenatal diagnosis have accelerated in the last decade changing our vision immensely. Data obtained from fetal genomic studies brought new insights to fetal medicine and by the advances in fetal DNA and RNA sequencing technology novel treatment strategies has evolved. Non-invasive prenatal diagnosis found ground in genetics and the results are widely studied in scientific arena. When Lo and colleges proved fetal genetic material can be extracted from maternal plasma and fetal DNA can be isolated from maternal serum, the gate to many exciting discoveries was open. Microarray technology and advances in sequencing helped fetal diagnosis as well as other areas of medicine. Today it is a very crucial prerequisite for physicians practicing prenatal diagnosis to have a profound knowledge in genetics. Prevailing practical use and application of fetal genomic tests in maternal and fetal medicine mandates obstetricians to update their knowledge in genetics. The purpose of this review is to assist physicians to understand and update their knowledge in fetal genetic testing from maternal blood, individualized prenatal counseling and advancements on the subject by sharing our experiences as İstanbul University Fetal Nucleic Acid Research Group.

## FREE FETAL NUCLEIC ACIDS IN MATERNAL BLOOD AND SERUM

Non-invasive prenatal diagnosis (NIPD) has become the most groundbreaking topics in medicine in the last decades. It can be applied at any time throughout pregnancy without the risk of fetal loss being the most prominent advantage. Its diagnostic sensitivity in some genetic diseases is close to invasive procedures and close to 100% in some chromosomal anomalies. Furthermore, NIPD enables research on fetal physiology avoiding any harm to fetus making it promising for future studies. Technology that enables fetal genetic analysis without invasive procedures will not be limited to this topic for sure. Presumably and progressively, the method will catch on in other branches of medicine evolving routes of diagnosis and therapy as we know it. There is already a new era in oncology where free tumor DNA analysis helping to diagnose cancer prior to metastasis. Due to its unique state, obstetrics and gynecology frontiered this approach similar to ultrasound and laparoscopy. We believe that obstetricians should comprehend the new revelations as soon as possible. On this note we founded İstanbul University Fetal Nucleic Acid Research Group as a collaboration of İstanbul University Cerrahpaşa Medical Faculty Obstetrics and Gynecology Ward and İstanbul University Molecular Biology and Genetics Ward.

The first and most important step in NIPD is the isolation of fetal nucleic acids. Mandel and Mateis observed cell free fetal DNA and RNA in both healthy and diseased subjects’ plasma in 1948^(1)^. However this discovery was not given proper credit at the time since it was not fully comprehended. Isolation of cell free fetal DNA (cffDNA) in maternal serum in 1997 by Lo et al. was the corner stone for NIPD^(2)^. Following the isolation of cffDNA numerous methods were developed to reveal genomic (DNA), transcriptomic (RNA) and proteomic functions via gene expression, gene quantification and genetic sequencing. Mounting these with advanced software technologies accelerated the progress in genetics in the last decade.

## CELL FREE FETAL DNA (CFFDNA)

cffDNA has been shown to exist in maternal spinal fluid, urine and intra-abdominal peritoneal fluid^(3)^. The ratio of cffDNA to free DNA in maternal serum serum is about 10% (3-19%)^(2)^ cffDNA, due to its relationship with placental microparticles is not hydrolised by circulatory nucleases and stays stable^(4)^. Ones the placental microparticals are cleared as in postpartum, is it quickly cleared from maternal circulation^(5)^. The source of cffDNA in maternal circulation is placenta, fetal hematopoietic cells and fetal DNA itself^(6)^. Physiological and clinical data reveal that most of free nucleic acids in circulation originate from placenta rather than fetal hematopoietic cells. cffDNA in maternal circulation can be detected as early as 28^th^ days after conception. Since fetoplacental circulation does not start at that time, cffDNA is more likely to be shed from trophoblasts rather than hematopoietic cells^(7)^. Maternal cell free DNA has 162 to 169 base pairs. Placental cffDNA on the other hand, has 143 base pairs since it does not contain 20 base pair extension to attach to nucleus. Wataganara et al. (2004), published strong evidences supporting placental origin of cffDNA when they examined first trimester elective pregnancy terminations. The study examined 134 first trimester elective pregnancy terminations, where 71 patients were surgically extracted and 63 were medically (misoprostol) induced. CffDNA levels in maternal blood following surgical extractions were significantly higher due to fetomaternal bleeding and destruction of trophoblastic villi. On the other hand, in medically induced terminations, cffDNA was positive in maternal blood 11 days after initiation of termination. Wataganara believed that this delay was caused by the residual placental tissue in uterus^(8)^.

Placental mass and circulatory fetal DNA levels do not correlate. Increased cffDNA in maternal blood is caused by placental hypoxia or trophoblastic destruction^(9,10)^. Even though most studies examined cffDNA in maternal blood, amniotic fluid is a good source of fetal DNA. Bianchi et al. showed that at 16-20 weeks of gestation, fetal DNA in amniotic fluid is 100 to 200 times the amount in maternal blood. It is hypothesized that cffDNA in amniotic fluid is more useful in illuminating fetal physiology. The introduction of cffDNA ignited many studies. The common terms used in molecular genetics were shown in Table 1.

## CELL FREE FETAL RNA (CFFRNA)

Poon et al. (2000) isolated Y chromosome specific mRNA transcript from maternal blood in patients carrying male fetuses and showed that cell free fetal RNA (cffmRNA) can be used as independent fetal nucleic acid indicator in maternal blood. The half-life of placental mRNA in maternal blood is approximately 14 minutes and can be detected in maternal plasma at 4 weeks^(11)^. In contrast to cffDNA, cffRNA levels do not increase as pregnancy proceeds^(12)^. Furthermore, detection of cffmRNA in maternal plasma is not associated with paternal polymorphism^(13)^. Extraction of gene transcripts specific to placenta such as βHcG (β subunit of human chorionic gonadotropin) and hPL (human placental lactogen) advocates that fetal RNA in maternal plasma comes primarily from placenta^(14)^. Fetal DNA and RNA circulating in maternal blood deflects the idea that placenta is an impermeable membrane.

## COLLECTING, TRANSPORTING AND STORING MATERNAL BLOOD

Following certain algorithms im collecting, transporting and storing maternal blood for NIPD avoids cffDNA destruction in samples. Since the ratio of cffDNA to maternal DNA is minute, proper transport and storage of samples is an important factor in reliability of the tests^(15)^. Maternal cell lysis due to warm environment releases excess amounts of maternal DNA to serum, which in turn alters NIPD results. Quantitative measurements of fetal DNA in fetal aneuploidy scanning would be less reliable in such cases. Currently maternal blood is collected in EDTA containing sampling tubes. In order to avoid maternal cell lysis, it is advised to centrifuge blood within 6 hours of sampling^(16,17)^. In 2013 Wong et al. compared efficiency of new blood collection tubes (BCT’s) to standard EDTA tubes. The study revealed that the new BCT’s contain chemicals which prevent maternal cell destruction and allow longer storage time. The study stored blood samples for two days and tested at from 4 to 37 Celsius degrees for one through 14 days. Results showed that maternal cell destruction started after 14 days which caused increased maternal DNA amounts. Currently BCT use is only approved for research purposes, however based on the results of this study, isolation of cffDNA from maternal blood can beneficiary using cell stabilizing chemicals^(16,17,18)^.

## ANALYSIS METHODS AND ADVANCES IN NON-INVASIVE MOLECULAR DIAGNOSIS

**Figure 1:**
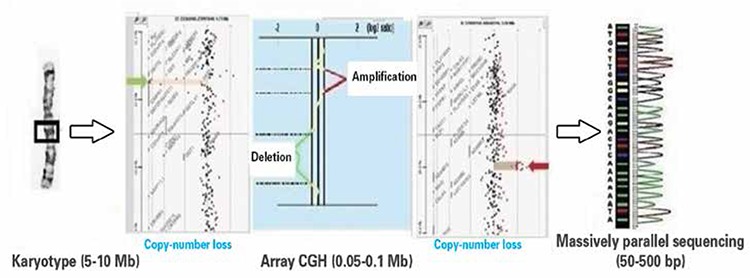
Comparing DNA region analysis by using conventional cytogenetic chromosome banding, array CGH and massively parallel sequencing methods

cffDNA and cffRNA technology is a rapidly improving area in NIPD. Indebted to advances in molecular analysis technology, cytogenetics has gained depth and detail. We summarized principles of mechanism and resolutions in Table 2 and Figure 1 respectively.

## EVALUATION OF ARRAY COMPARATIVE GENOMIC HYBRIDIZATION AND MICROARRAY METHODS

Array comparative genomic hybridization (aCGH) and microsequencing are innovative technologies with different clinical applications. Both methods give high resolution analysis (0.05-0.1 Mb) of nucleic acids and commonly referred as microarray method in literature. Main difference lies in clinical application. Genetic material is identical in every cell with rare exceptions such as somatic mutations and placental mosaicism. Array CGH compares two different genomes for DNA repetition numbers. It is primarily used for diagnosing micro-deletion and micro-duplication syndromes by calculating number of repetitions on DNA segments.

Microarray method usually applies to RNA. Gene expression profile varies according to the cellular phase and environment. Microarray is simply the tool to describe these conditions. In short, microarray reveals alterations in gene expression (transcriptomic microarray). Alterations in gene expression in certain conditions are monitored to acquire clues about the proteins coded by specific genes. Microarray method is used to monitor gene expression patterns in various physiological or pathological events.

## FETAL TRANSCRIPTOMIC ANALYSIS

Fetal transcripts in maternal blood are extremely scarce since most of the genetic material is of maternal origin. It has been hypothesized that^(40)^ amniotic supernatant contains enough free fetal RNA to reveal gene expression patterns on human growth. The study concluded that fetal gene expression modifies according to geststional age and sex. Amniotic transcriptomes from trisomy 21 and 18 subjects were compared healthy controls for the same gestational age and sex where both aneuploidies showed hundreds of statistically significant genetic alterations among which very little was chromosome mapped. Tree hundred eleven genes out of 414 were unalike in trisomy 21 subjects where only 5 of those were localized on chromosome 21. Similarly, 251 genes out of 352 were miscorrelated in trisomy 18 subjects and only 7 of those were located on chromosome 18^(41)^. These findings prove that pathologies in aneuploidic fetus does not solely result from the extra chromosome ordered gene amounts.

## CLINICAL APPLICATIONS

### Non-invasive Diagnosis of RH Disease

Hemorrhagic disease of the newborn (erythroblastosis fetalis) is a disorder where paternal erythrocytes carry rhesus antigen and maternal cells do not. When the mother is encountered with RH positive antigens, the antibodies produced to these antigens can travel through placenta to aggregate RH positive fetal cells. Detecting fetal RH antigens from free fetal DNA in maternal blood is approved by International Blood Type Reference Laboratories since 2001 after successful preclinical trials^(50)^. However, the large scale studies in France and Netherlands revealed some obstacles in non-invasive RHD tests. False negative results are usually caused by low fetal DNA counts due to either biological (very early pregnancy) or technical (low extraction rate) problems. In order to overcome these biases, recent studies advised the introduction of additional PCR methods; SRY region in y chromosome, differentially methylated placental DNA marker RASSF1A or paternal genetic markers as secondary internal controls. Usually false positive results occur in African originated RH negative subjects in the presence of two RHD variants; RHD pseudo-gene or RHD-CE-Ds hybrid sequences. Following the description of these variants, specific primers and probes were manufactured to detect and avoid false positive amplification^(51,52)^. We studied 70 RH incompatible patients and diagnosed 48 RHD positive, 19 RHD negative and 3 RHD variant fetuses. All non-invasive tests were confirmed postnatally^(50,51)^.

## NON-INVASIVE DIAGNOSIS OF SEX LINKED DISEASES

PCR multiplication of cffDNA in maternal blood is now an alternative to invasive procedures for fetal sex determination. It can be preferred in X linked diseases, ambiguous genitalia in ultrasound exam or in cases of congenital adrenal hyperplasia trait where early administration of steroids in order to prevent female fetus from masculinisation is crucial. The statistical power for non-invasive fetal sex determination was the subject of 57 studies and meta-analysis of 80 different data sets on 6541 singleton pregnancies, concluding 95.4% specifity and 98.1% sensitivity. The main parameters affecting non-invasive sex determination results were gestational age and DNA amplification method. Nevertheless, England endorsed NIPD of fetal sex in an attempt to decrease the frequency of invasive testing for sex linked diseases^(53)^. As İstanbul University Fetal Nucleic Acid Research Group, we determined 30 patients prenatally confirming the sex after birth.

## NON-INVASIVE DIAGNOSIS IN SINGLE GENE DISORDERS

The sensitivity and specifity of NIPD in the first trimester is still limited because maternal and fetal DNA are aliened in serum^(54)^. Enrichment of fetal DNA in maternal plasma can increase the precision and enable further analysis for diseases such as achondroplasia, hemoglobinopathies, congenital adrenal hyperplasia, cystic fibrosis, Huntington Disease and myotonic dystrophia^(55)^.

One of the common single gene disorders constituting a public health issue in Turkey is thalassemia. There are 400 mutations in the thalassemia gene described up-to-date^(56)^. The diagnosis involves molecular methods, mainly by DNA extraction. In order to diagnose thalassemia in utero; amniotic fluid, chorion villus sampling or fetal blood were obtained invasively causing 0.5 to 1 percent fetal loss. Replacing invasive testing with NIPD methods is highly advocated and becoming widespread as new data keeps coming^(57,58)^.

## NIPD OF FETAL ANEUPLOIDIES

Compared to other prenatal disorders, diagnosis of fetal aneuploidies requires more delicate technical methods since there is not a single mutation or defect. The studies revealed that cffDNA in maternal plasma and serum increase in Trisomy 21 and 13^(59,60,61)^ but remain constant in Trisomy 18^(62)^. Two studies concluded that there is no correlation between cffDNA levels in maternal plasma or serum with respect to aneuploidies^(60,61,62,63)^. The common opinion that there is a strongly probable correlation between increased cffDNA and aneuploidies resulted in using the cffDNA levels in second trimester in combination with quadruple test. This approach increased detection rate of Down Syndrome in second trimester from 81% to 86%^(64)^.

Epigenetic modifications are other proposed markers for prenatal diagnosis. Epigenetic modifications ie. DNA methylations, are alterations in gene expression or phenotype without modification in DNA sequence^(65)^. Discovery of DMR’s (Differentially Methylated Regions); which simply are dissimilar methylated maternal and fetal DNA fragments, enabled to differentiate maternal and paternal alleles in the fetus^(66)^. Epigenetic-genetic chromosome quantification method uses holocarboxylase synthetase (HLCS) gene putative promoter. HLCS gene is located on chromosome 21 and assures diagnosis of Trisomy 21 in both male and female subjects^(67)^. Maspin is the first universal fetal DNA marker in maternal plasma. It is a placenta specific epigenetic marker which is found in methylated state in maternal leucocytes whereas hypomethylated in placenta^(68)^. Maspin, also known as SERPINB5 gene, proves that placental and maternal cells are differentially methylated. This discovery enhanced utilizing epigenetic markers for the diagnosis of Trisomy 18^(68,69)^ and searching for novel fetal markers for Trisomy 21^(70,71)^. 114 regions on chromosome 21 were studied, resulting in discovery of 22 DMR’s^(72)^. Latest researches used methylated DNA immune precipitation (MeDiP) and Rt-PCR for non-invasive prenatal diagnosis of trisomy 21^(73)^. They identified fetal DMR’s methylation rate from maternal blood, and comparing it with 26 euploid controls, they were able to diagnose Trisomy 21 successfully. There are two important advantages to MeDiP compared to other epigenetic methods; the use of DMRs containing cutting regions and sodium bisulphate (methylation sensitive enzyme resulting in DNA lysis) is eliminated^(74)^.

Microfluidic chips are developed in order to compare Trisomy 21 cell line with healthy human cell line^(29)^. GAPDH (Glyceraldehyde Phosphate Dehydrogenase) locus levels on chromosome 12 were compared with amyloid gene sequence on chromosome 21. Even though the samples constituted only 10% of total material (sample/number), digital PCR could differentiate between healthy and aneuploidic subjects reliably proving it to be more sensitive than RT-PCR and fluorescent QF-PCR^(23)^. Digital PCR studies enables diagnosis of fetal aneuploidies using cffDNA from maternal blood. Same researchers could appropriately diagnose fetal trisomies from 24 amniotic fluid samples and 16 chorion villus samples using microfluidic digital PCR method^(75)^.

Lo et al. detected fetal aneuploidies using cffDNA and cffmRNA digital PCR method^(22)^. Two digital PCR methods were developed for the NIPD of fetal aneuploidies. The first one is based on PLAC4 Mrna SNP method. PLAC4 is gene on chromosome 21 coding a specific placental mRNA. The allele rate in maternal blood of this particular mRNA can detect Trisomy 21 with 90 to 96.5% sensitivity^(76)^. The second regimen relies on dosage method where comparison of two different loci dosages on chromosome 21 and chromosome 1 is studied. This regimen allows the detection of fetal aneuploidies even when there is minute amounts of (25%) trisomic DNA.

Improvements in digital PCR technique had great impact on DNA sequencing technology. Analysis of cffDNA in maternal plasma by MPSS-next generation sequencing-has been reported in two different studies in 2008^(45,77)^. Following these two reports, cffDNA in maternal plasma of Trisomy 13, 18 and 21 were sequenced with high sensitivity and specifity^(78,79)^.

cffDNA results in maternal plasma obtained by MPSS regimen are listed in terms of Z-scores. Z-score basically reflects the measured value’s standard deviation in multiples from median measurements. In healthy pregnancies, z-score distribution is similar to Gaussian curve which is under 3 for euploid fetuses. Z-score above 3 indicates Trisomy 21 with 99.87% probability^(48)^.

Chiu et al. are the first to validate the clinical applications of MPSS sequencing for cffDNA in maternal plasma^(79)^. Study group consisted of 314 subjects under high risk for Trisomy 21. 2-plex sequencing yielded results with 100% sensitivity and 97.9% specifity. 8-plex sequencing were carried out in 753 maternal plasma samples revealing 79.1% detection rate and 98.9% specifity for Trisomy 21. Ehrich et al. studied 499 maternal plasma samples with MPSS and reported 100% sensitivity and 99.7% specifity^(78)^. Sehnert et al. studied 119 cffDNA samples from maternal plasma by MPSS and detected Trisomy 18 in addition to Trisomy 21^(80)^ with 100% accuracy (8/8 for Trisomy 18 and 13/13 for Trisomy 21. Chen et al. examined 392 maternal plasma samples by MPSS for non-invasive prenatal diagnosis of Trisomy 13 and 18^(81)^. This study determined the correct GC ratio by using non-repeat-masked reference genomes and bioinformatics. The sensitivity and specifity for Trisomy 13 was 100% and 98.9% respectively (25 out of 25). Similarly, the sensitivity and specifity for Trisomy 18 was found 91.8% and 98% respectively (34 out of 37). Although these findings are very promising and exiting, in the previous study DNA amounts were very small size and DNA sequencing was not carried out in a non-CLIA certified laboratory. Palomaki et al. performed clinical validations is order to overcome these bias^(82)^. These scientists diagnosed Trisomy 21 by using “next generation sequencing” in 212 pregnancies carrying Trisomy 21 fetuses matching to 1484 euploid controls. They had 0.2% false positive rate and 98.6% detection rate. The same researchers used MPSS to identify Trisomy 13 and 18 and published 100% and 91.7% detection rate for Trisomy 18 and Trisomy 13 respectively^(83)^ and false positive rate 0.28 and 0.97% respectively.

Currently cffDNA analysis by MPSS is still vague in terms of prenatal diagnosis. Whether NIPD by MPSS should be carried out prior to invasive procedures in high risk pregnancies or replace all screening tests requires further evaluation. Evidently decreases in MPSS costs would aid the process. International Society for Prenatal Diagnosis (ISPD) published a guideline for screening strategies for aneuploidies in 2011. According to this guideline, non-invasive prenatal screening and diagnosis by fetal nucleic acids in maternal circulation is not yet validated and therefore not to be recommended in clinical practice^(84)^. In response to this opinion, Palomaki et al. prepared a report on MPSS sequencing in Trisomy 21 fetuses, concluding that advanced screening tests and confirmation of MPSS positive results by invasive testing should be carried out^(85)^. In light of these extensive scientific reports, MPSS is now used in US and Europe as method for aneuploidy diagnosis.

MPSS sequencing studies reveal over 99% sensitivity and specifity for the diagnosis of autosomal trisomies which are also feasible in multiple pregnancies. At this point, we should explore if the false positive results are caused by the method itself or the genetic material. Considering the rare occasions such as somatic mutations and placental mosaicism, and the knowledge we have so far obtained by invasive prenatal procedures, there are two possible reasons for false positivity. Infrequently, the necessity for additional invasive procedures arises. For instance, when confirmed by amniocentesis or fetal blood sampling, the 1-2% mosaicism rate in CVS reduces to 0.15%. cffDNA analyzed in MPSS is of placental origin. Therefore, there will be false positive results due to placental mosaicism (it seems impossible to evade false positive results due to placental mosaicism). In case of a pathologic finding in MPSS, it is advocated to keep placental mosaicism in mind and chose a method other than CVS.

The false negative outcomes in MPSS are mostly disciplinary. When MPSS analyses first started, EDTA containing sample collection tubes were used and there was not a consensus on required amounts of cffDNA or minimum base pair reads (sequence reads; Meb=M). The false negative cases in all series were from initiation stages. As the learning curve proceeded, BCT’s (blood collection tubes) were introduced by Wong et al in 2013^(18)^. These tubes enabled analysis of cffDNA without the influence of maternal maternal DNA. In consequence of this advancement, it is advised to have a minimum of 4% fetal DNA and 10 Meb (10 million) base pair reads for correct outcomes. Whether these criteria help diminish false negativity or not, is to be exposed in the near future.

## CONCLUSION

RT-PCR, dPCR, Array CGH, microarray and MPSS methods are now available for research and NIPD. Merits to these methods, prenatal diagnosis of some fetal disorders is applied to clinical practice. 

Some paternal inherited diseases such as achondroplasia, fetal sex, Rh factor and various single gene disorders can be diagnosed by NIPD. Array technology (microsequencing) detects quantitative and structural chromosome abnormalities causing genomic alterations with high accuracy.

Currently, it is possible to claim over 99% sensitivity and specifity in diagnosis of fetal autosomal trisomies, Monosomy X, sex chromosome abnormalities, and microdeletion syndromes (DiGeorge, Cri-du-chat, 1p36, Angelman, Prader Willi) analyzing cffDNA from maternal blood via MPSS technology. It is advised to confirm positive test results affected fetus-with invasive procedures other than CVS. Whether NIPD by MPSS method should be done in high risk pregnancies prior to invasive procedures, or replace the current screening tests is to be decided in the near future. Clearly, a decrease in MPSS costs will affect the diagnosis algorithms. Scientifically speaking, NIPD by MPSS with its 99% accuracy in autosomal trisomies, Monosomy X and some microdeletion syndromes will reduce invasive procedure rates by 90%. NIPD will also endorse high detection rates in invasive tests and avoid “amniocentesis anxiety” in low risk patients (high risk in screening tests, advanced maternal age, minor marker presence in ultrasound).

## Figures and Tables

**Table 1:**
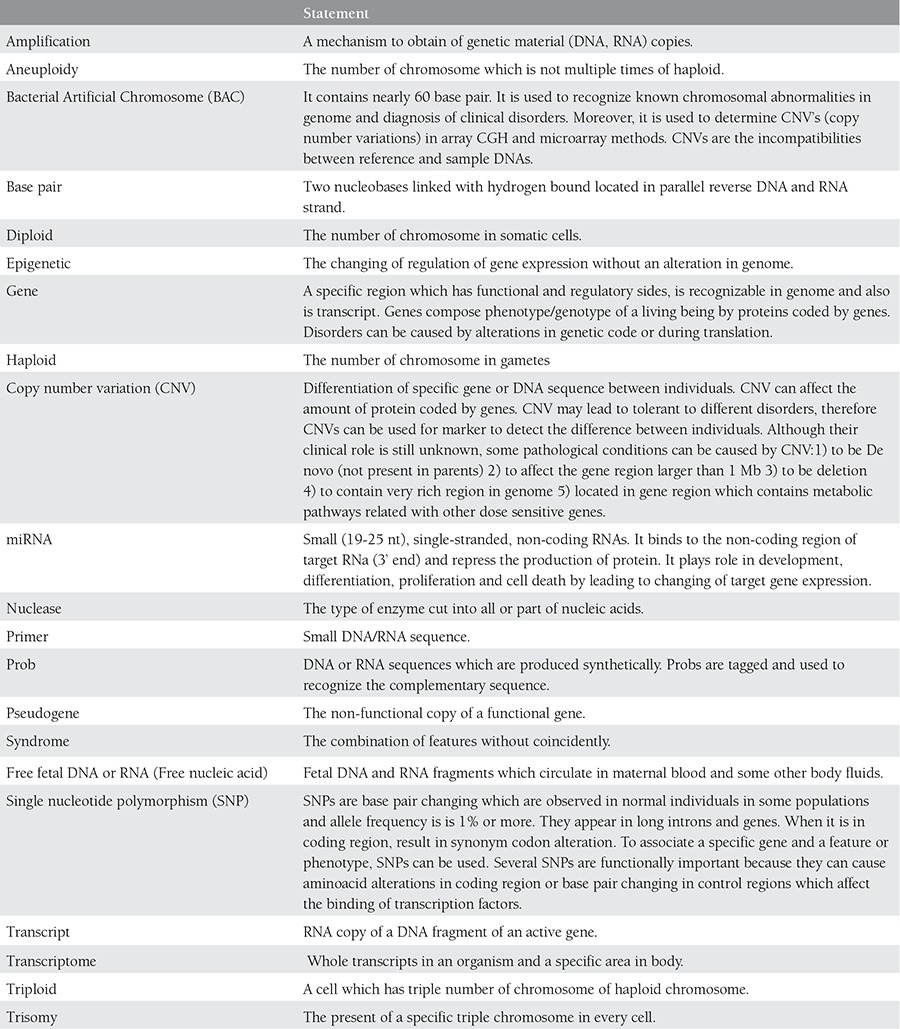
Several terms used in molecular genetics

**Table 2:**
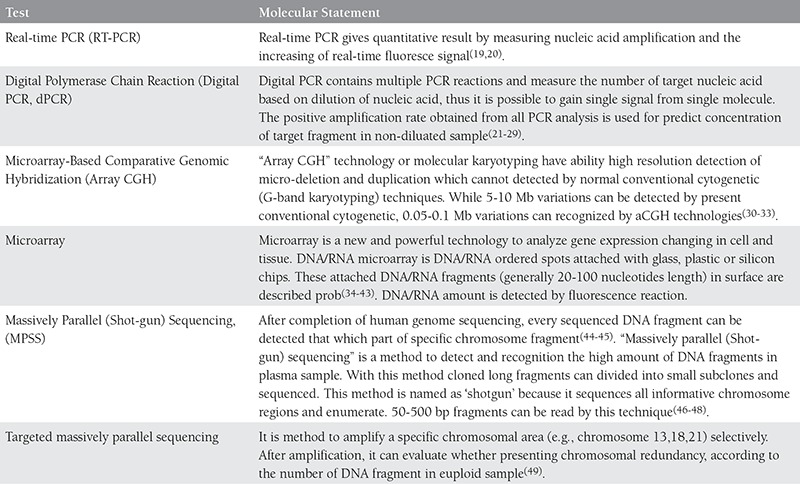
Molecular tests used for fetal diagnosis and treatment
